# Supplementary material for: A Likelihood Approach for Real-Time Calibration of Stochastic Compartmental Epidemic Models
Source: PLoS Comput Biol. 2017 Jan 17;13(1):e1005257. doi: 10.1371/journal.pcbi.1005257 (PMC5240920; doi:10.1371/journal.pcbi.1005257)
Supplement: S1 File — (TAR.GZ) [file pcbi.1005257.s014.tar.gz › HSPH_Online-SI-Revision/MSS10/n10-extreme/n10-extreme_NEW_table-8.pdf]

1	R0	{2.68692, 12.5884, 1.}	{2.29614, 10.3721, 0.62}	{{1.47608, 5.58477, 0.}, {2.7811, 15.2661, 2.}}	{16.8566, 29.2041, 74.}	inf	21.	26.2754	{5.18251, 42.5256}	58.3679	0.259734	newly	13.	30.14	{7., 59.}	148.769	0.373179	Reff	2.6759	2.2851	{1.47233, 2.76114}	16.8235	ar	9089.	8082.53	{5735., 9267.}	12.0853
2	R0	{1.79802, 12.5412, 1.}	{1.2152, 3.73909, 0.43}	{{1.06738, 2.19254, 0.}, {1.44762, 6.00596, 2.}}	{34.3998, 72.4414, 99.}	inf	125.	43.1126	{8.43544, 76.2366}	69.6444	0.650061	newly	89.	97.69	{24., 176.}	51.7416	0.269344	Reff	1.74857	1.19076	{1.04564, 1.41871}	33.8301	ar	7412.	2578.62	{259., 5491.}	66.5483
3	R0	{2.6174, 10.1105, 0.1}	{1.91534, 13.8836, 0.35}	{{1.13227, 6.87793, 0.}, {2.77394, 19.6025, 1.}}	{29.6758, 48.7181, 394.}	inf	4.	3.05597	{1.23981, 6.00176}	51.6259	0.28794	newly	3.	3.74	{0., 12.}	117.333	0.597892	Reff	2.61609	1.91451	{1.13194, 2.77286}	29.6711	ar	9109.	5227.46	{3., 9249.}	43.2451
4	R0	{2.27532, 14.3149, 0.1}	{2.30791, 14.2282, 1.19}	{{1.87584, 9.63533, 0.}, {2.86501, 18.4249, 2.}}	{16.6104, 26.4107, 1116.}	inf	174.	182.572	{94.7708, 285.488}	32.8944	0.183217	newly	107.	118.24	{82., 158.}	25.1589	0.110504	Reff	2.20251	2.2288	{1.82632, 2.75317}	16.0363	ar	8478.	8267.44	{7496., 9336.}	10.0106
5	R0	{2.96649, 18.0674, 2.}	{2.0309, 10.2134, 1.22}	{{1.24751, 3.26445, 0.}, {2.8833, 17.5057, 2.}}	{31.6347, 44.0773, 49.}	inf	107.	53.0596	{11.3536, 94.9129}	51.2899	0.432948	newly	64.	56.13	{25., 91.}	36.3281	0.200457	Reff	2.92051	2.00845	{1.24011, 2.83499}	31.3715	ar	9476.	6880.4	{3743., 9334.}	27.3953
6	R0	{2.94778, 16.0002, 0.1}	{1.95855, 7.17213, 0.44}	{{1.28596, 3.83841, 0.}, {2.74056, 12.2271, 2.}}	{33.6672, 55.1747, 480.}	inf	56.	33.3319	{10.2463, 62.2182}	47.5632	0.358472	newly	41.	56.16	{17., 101.}	64.9268	0.242887	Reff	2.92125	1.94464	{1.27833, 2.71897}	33.5477	ar	9321.	7064.92	{3867., 9217.}	24.2734
7	R0	{2.17562, 11.7184, 1.}	{1.49613, 4.61435, 0.35}	{{1.06738, 1.89883, 0.}, {2.58092, 12.5436, 2.}}	{38.279, 69.7208, 97.}	inf	23.	13.9684	{3.1857, 23.6772}	46.415	0.340857	newly	18.	47.37	{9., 95.}	187.167	0.39545	Reff	2.16844	1.49231	{1.06528, 2.57155}	38.2258	ar	8345.	4014.43	{34., 9014.}	54.647
8	R0	{2.7904, 17.8966, 2.}	{2.054, 9.65376, 0.68}	{{1.24751, 2.5364, 0.}, {2.86656, 18.1089, 2.}}	{27.8117, 46.8977, 66.}	inf	98.	68.1995	{23.6628, 119.631}	40.0316	0.275601	newly	46.	82.43	{38., 136.}	87.5	0.252351	Reff	2.74575	2.0256	{1.23606, 2.8129}	27.5948	ar	9285.	7244.72	{3643., 9346.}	22.2092
9	R0	{2.51574, 16.376, 1.}	{2.3575, 11.6614, 0.58}	{{1.93419, 8.31782, 0.}, {2.68803, 17.1903, 2.}}	{9.94936, 30.6068, 66.}	inf	112.	86.9371	{51.6924, 119.03}	27.3834	0.151711	newly	55.	82.76	{53., 116.}	54.9455	0.178532	Reff	2.47171	2.32168	{1.90383, 2.63164}	9.82815	ar	8937.	8582.58	{7651., 9049.}	5.15587
10	R0	{2.56405, 18.1787, 2.}	{1.39203, 4.4798, 0.27}	{{1.06738, 2.19254, 0.}, {1.87584, 10.2502, 2.}}	{46.221, 75.4862, 90.5}	inf	124.	37.0849	{7.30955, 67.301}	70.4049	0.66721	newly	69.	83.05	{18., 149.}	59.4638	0.275047	Reff	2.51226	1.37516	{1.05696, 1.85132}	45.7755	ar	9005.	4002.44	{171., 7395.}	55.7026
11	R0	{2.475, 17.1709, 1.}	{1.84435, 14.2312, 1.22}	{{1.48482, 9.52864, 1.}, {2.20181, 18.765, 2.}}	{25.4809, 23.594, 40.}	inf	144.	86.5302	{41.4307, 129.48}	39.9659	0.267061	newly	77.	53.38	{33., 65.}	35.7922	0.197048	Reff	2.41461	1.80902	{1.45881, 2.15628}	25.0803	ar	8870.	7203.75	{5862., 8472.}	18.7852
12	R0	{2.14974, 11.8619, 0.1}	{2.16242, 13.1607, 1.17}	{{1.40191, 6.71734, 0.}, {2.86501, 18.4249, 2.}}	{17.7682, 31.611, 1100.}	inf	45.	44.4502	{13.9749, 72.1024}	35.7062	0.180099	newly	37.	32.06	{18., 47.}	27.5135	0.147672	Reff	2.13147	2.14337	{1.39436, 2.84175}	17.6309	ar	8414.	7903.68	{5067., 9308.}	12.1096
13	R0	{2.20385, 14.4767, 0.1}	{1.90673, 11.7803, 0.1}	{{1.67732, 9.44073, 0.}, {2.30108, 15.555, 0.}}	{16.2764, 23.8973, 182.}	inf	171.	110.914	{69.0558, 171.844}	36.286	0.218522	newly	105.	88.07	{57., 111.}	23.1524	0.116026	Reff	2.13685	1.85929	{1.63653, 2.2324}	15.8313	ar	8347.	7457.46	{6676., 8627.}	12.3407
14	R0	{2.35671, 13.8357, 1.}	{2.31514, 9.3796, 0.34}	{{1.737, 4.56746, 0.}, {2.9884, 12.8326, 1.}}	{17.6334, 33.0533, 74.}	inf	81.	64.7666	{23.1826, 103.81}	35.2866	0.229855	newly	53.	80.2	{34., 121.}	65.2453	0.217786	Reff	2.32631	2.28778	{1.71735, 2.94201}	17.428	ar	8719.	8255.54	{6847., 9371.}	10.334
15	R0	{1.91368, 10.1082, 1.}	{2.32592, 17.6267, 2.03}	{{2.04423, 14.1631, 2.}, {2.48947, 19.255, 2.}}	{22.152, 74.3803, 103.}	inf	100.	127.047	{86.619, 160.15}	33.6842	0.126741	newly	95.	66.08	{47., 84.}	30.8842	0.172651	Reff	1.87579	2.27319	{1.99485, 2.43164}	21.7618	ar	7486.	8615.46	{8031., 8964.}	15.2725
16	R0	{2.48727, 14.882, 0.1}	{2.58199, 12.5114, 0.37}	{{2.14754, 8.13253, 0.}, {2.96947, 18.0404, 1.}}	{11.1022, 24.3076, 404.}	inf	30.	31.8869	{12.5329, 49.7188}	36.8913	0.169725	newly	19.	31.37	{12., 60.}	79.2105	0.23528	Reff	2.47508	2.56861	{2.14224, 2.95211}	11.023	ar	8894.	8943.79	{8329., 9394.}	4.15055
17	R0	{2.46795, 13.1915, 0.1}	{1.82031, 7.75216, 0.88}	{{1.06738, 2.19254, 0.}, {2.86095, 15.7308, 2.}}	{31.84, 52.6534, 890.}	inf	43.	12.6945	{3.56835, 23.2954}	70.4779	0.630334	newly	22.	26.37	{6., 54.}	84.1364	0.324095	Reff	2.45487	1.81587	{1.066, 2.85378}	31.7406	ar	8796.	5932.38	{511., 9264.}	34.6294
18	R0	{2.96603, 16.7543, 0.1}	{2.16572, 8.96224, 0.43}	{{1.53501, 4.02511, 0.}, {2.73634, 13.3345, 1.}}	{27.0126, 46.6249, 462.}	inf	59.	55.5981	{20.7801, 86.8831}	33.342	0.179417	newly	37.	71.14	{39., 107.}	98.2703	0.280804	Reff	2.93429	2.14243	{1.52551, 2.70273}	27.0037	ar	9394.	7974.74	{6231., 9199.}	15.1235
19	R0	{2.25216, 9.90465, 0.1}	{2.06688, 11.969, 0.74}	{{1.28596, 5.71232, 0.}, {2.86001, 18.6082, 2.}}	{20.5233, 39.1759, 738.}	inf	7.	7.10056	{2.53262, 13.3907}	47.1413	0.225465	newly	5.	7.41	{1., 16.}	92.6	0.340417	Reff	2.24946	2.06429	{1.28442, 2.85471}	20.5117	ar	8631.	7107.91	{240., 9288.}	21.1337
20	R0	{2.70837, 18.1923, 0.1}	{2.20112, 13.0684, 0.99}	{{1.47608, 6.71734, 0.}, {2.86501, 18.8445, 2.}}	{21.1071, 29.8981, 938.}	inf	85.	47.688	{18.9466, 77.3257}	44.9559	0.309757	newly	45.	35.64	{18., 53.}	30.3556	0.171851	Reff	2.67506	2.18148	{1.46874, 2.83447}	20.8663	ar	9183.	8086.76	{5790., 9335.}	12.4863
21	R0	{2.30288, 15.5877, 1.}	{2.49168, 16.6842, 0.24}	{{2.11132, 12.644, 0.}, {2.93978, 19.9817, 1.}}	{12.9354, 16.481, 78.}	inf	145.	161.954	{113.337, 209.576}	22.7595	0.0911245	newly	76.	92.42	{63., 118.}	28.1053	0.10656	Reff	2.2437	2.42255	{2.06177, 2.85045}	12.6165	ar	8674.	8827.55	{8189., 9375.}	4.70152
22	R0	{2.82208, 10.7544, 0.1}	{1.86624, 11.4626, 0.64}	{{1.12882, 5.71232, 0.}, {2.86501, 18.7084, 2.}}	{34.8727, 33.501, 652.}	inf	4.	3.92956	{1.44435, 6.84585}	48.4959	0.22691	newly	9.	5.9	{1., 8.}	88.4444	0.647482	Reff	2.82039	1.86506	{1.12841, 2.86295}	34.8673	ar	9333.	5093.67	{4., 9288.}	45.5081
23	R0	{1.69923, 10.1366, 1.}	{1.24947, 3.3496, 0.12}	{{1.08063, 2.5364, 0.}, {1.28596, 3.98513, 0.}}	{26.7597, 67.7436, 94.}	inf	114.	40.2061	{9.07845, 67.2612}	64.8492	0.560822	newly	88.	111.01	{23., 200.}	62.8977	0.277204	Reff	1.65454	1.22574	{1.05896, 1.26239}	26.2059	ar	7055.	3407.55	{1125., 4319.}	51.9347
24	R0	{2.64944, 19.054, 1.}	{2.53295, 14.538, 0.01}	{{2.14747, 10.7247, 0.}, {2.6365, 15.4074, 0.}}	{6.08007, 23.7009, 99.}	inf	333.	317.081	{247.193, 377.289}	11.0593	0.05356	newly	143.	215.08	{180., 252.}	50.4196	0.173752	Reff	2.50266	2.39589	{2.05519, 2.49678}	5.9277	ar	9147.	8916.96	{8386., 9155.}	2.88925
25	R0	{2.53191, 10.9214, 0.1}	{1.65885, 13.4586, 0.69}	{{1.21457, 8.26611, 0.}, {2.08951, 19.0665, 2.}}	{34.4824, 36.7532, 680.}	inf	6.	7.28028	{2.2257, 13.6486}	57.5368	0.227884	newly	9.	4.91	{1., 9.}	55.4444	0.418442	Reff	2.52811	1.65608	{1.21314, 2.08672}	34.4935	ar	9009.	5630.21	{24., 8211.}	37.5046
26	R0	{1.89155, 6.76944, 0.1}	{2.37497, 5.93198, 0.24}	{{1.24751, 2.19254, 0.}, {2.99088, 10.1535, 1.}}	{38.4069, 38.0079, 312.}	inf	21.	21.9087	{3.91811, 41.8617}	52.0392	0.281235	newly	28.	86.02	{15., 193.}	226.714	0.457762	Reff	1.88512	2.36625	{1.24566, 2.97899}	38.333	ar	7348.	7656.94	{1428., 9399.}	30.1298
27	R0	{2.63149, 15.6979, 0.1}	{2.36537, 11.9039, 0.29}	{{1.87584, 8.12562, 0.}, {2.90165, 17.1903, 1.}}	{14.4054, 27.5801, 338.}	inf	43.	28.0955	{11.2161, 42.094}	38.9757	0.254464	newly	23.	26.27	{12., 41.}	39.4348	0.154399	Reff	2.61465	2.35346	{1.86813, 2.88547}	14.3185	ar	9104.	8472.9	{7689., 9337.}	7.93651
28	R0	{2.85141, 12.6992, 1.}	{2.03339, 6.27769, 0.38}	{{1.28596, 2.5364, 0.}, {2.8702, 12.1446, 2.}}	{29.3943, 56.2942, 98.}	inf	11.	9.13742	{2.33188, 18.955}	49.6524	0.294571	newly	9.	41.92	{3., 77.}	394.444	0.488402	Reff	2.84685	2.03035	{1.28444, 2.86696}	29.3791	ar	9317.	6683.76	{33., 9287.}	28.3639
29	R0	{2.54244, 12.9085, 0.1}	{2.31763, 16.4454, 0.11}	{{1.97176, 13.0262, 0.}, {2.66225, 19.9254, 0.}}	{11.3987, 30.0812, 190.}	inf	35.	33.8773	{17.5206, 47.2439}	31.0801	0.149131	newly	22.	19.45	{10., 30.}	32.4091	0.165163	Reff	2.52846	2.30493	{1.96161, 2.64563}	11.325	ar	9034.	8569.31	{7836., 9116.}	5.58457
30	R0	{2.10982, 11.7636, 1.}	{2.38483, 12.2719, 0.36}	{{1.737, 5.43578, 0.}, {2.93488, 18.4249, 1.}}	{22.5089, 38.181, 64.}	inf	84.	116.017	{49.6467, 180.787}	56.4191	0.209065	newly	65.	104.97	{65., 146.}	65.	0.204061	Reff	2.07501	2.33577	{1.71015, 2.86881}	21.9494	ar	8224.	8393.54	{6988., 9377.}	11.6783
31	R0	{2.76312, 15.5396, 0.1}	{2.51695, 8.34006, 0.22}	{{1.53501, 4.06661, 0.}, {2.99092, 10.5991, 1.}}	{14.4492, 46.3303, 288.}	inf	70.	56.2631	{20.1019, 86.0749}	31.7981	0.197592	newly	46.	96.09	{41., 146.}	116.804	0.314611	Reff	2.73438	2.49358	{1.52465, 2.95729}	14.3849	ar	9220.	8475.95	{6108., 9410.}	9.36627
32	R0	{2.21727, 15.5439, 0.1}	{2.24033, 18.4748, 0.}	{{2.23915, 18.4504, 0.}, {2.27582, 18.9086, 0.}}	{2.5348, 19.5563, 100.}	inf	175.	174.583	{151.918, 194.231}	7.93436	0.0347879	newly	111.	83.36	{68., 98.}	24.9369	0.128358	Reff	2.14809	2.1705	{2.16355, 2.20894}	2.52456	ar	8359.	8503.98	{8409., 8638.}	2.59457
33	R0	{2.72912, 10.8159, 0.1}	{2.11555, 13.2052, 1.23}	{{1.28596, 3.98513, 0.}, {2.86501, 18.4249, 2.}}	{24.0147, 42.6442, 1180.}	inf	10.	14.4103	{4.13533, 25.0064}	76.6813	0.278482	newly	16.	15.46	{2., 32.}	80.75	0.440139	Reff	2.72393	2.11035	{1.28426, 2.85666}	24.018	ar	9196.	7400.05	{3841., 9280.}	19.9177
34	R0	{2.20928, 10.1308, 1.}	{2.14896, 3.30591, 0.02}	{{1.39162, 1.89883, 0.}, {2.99281, 4.56746, 0.}}	{23.9037, 67.3678, 98.}	inf	22.	17.3718	{3.03854, 33.2133}	47.7484	0.319198	newly	14.	225.04	{20., 423.}	1509.14	0.918544	Reff	2.20155	2.14219	{1.38655, 2.98293}	23.8681	ar	8388.	7484.15	{3995., 9377.}	18.7622
35	R0	{2.10103, 15.2673, 2.}	{2.30829, 13.6261, 1.24}	{{1.68297, 7.5059, 0.}, {2.86095, 19.9929, 2.}}	{20.1471, 23.7875, 38.}	inf	193.	137.339	{77.5189, 198.757}	32.1045	0.197024	newly	113.	98.88	{73., 124.}	18.9204	0.0935412	Reff	2.038	2.24989	{1.64882, 2.77687}	20.0823	ar	8264.	8329.98	{6883., 9330.}	10.6203
36	R0	{2.74914, 19.2775, 1.}	{1.92721, 12.5509, 0.42}	{{1.67732, 9.44073, 0.}, {2.46477, 18.6082, 1.}}	{29.8976, 34.8933, 66.}	inf	143.	82.9285	{47.609, 127.126}	42.1739	0.271	newly	67.	58.46	{42., 76.}	20.	0.102687	Reff	2.68618	1.89383	{1.64918, 2.40853}	29.4974	ar	9301.	7520.94	{6748., 8817.}	19.1384
37	R0	{2.78161, 16.0136, 0.1}	{2.43713, 15.9503, 0.3}	{{1.93539, 11.1369, 0.}, {2.93978, 18.8857, 1.}}	{15.1404, 17.5224, 348.}	inf	38.	26.1613	{9.03976, 41.0895}	37.1136	0.244805	newly	25.	16.99	{6., 27.}	45.32	0.281908	Reff	2.76659	2.42654	{1.92777, 2.92835}	15.0696	ar	9240.	8612.33	{7863., 9364.}	7.46851
38	R0	{2.4454, 8.33501, 0.1}	{1.9493, 12.0959, 0.62}	{{1.14884, 7.20429, 0.}, {2.64721, 16.7932, 2.}}	{24.4326, 53.7418, 626.}	inf	3.	5.46509	{1.54612, 11.1524}	107.668	0.294465	newly	9.	5.61	{0., 13.}	68.5556	0.593975	Reff	2.44369	1.94737	{1.14816, 2.64217}	24.4279	ar	8853.	6332.75	{16., 9070.}	29.8417
39	R0	{2.9154, 12.3861, 0.1}	{1.97592, 14.1492, 0.52}	{{1.21457, 8.33653, 0.}, {2.81891, 19.1478, 1.}}	{32.4523, 30.5246, 538.}	inf	14.	4.54012	{1.66361, 8.14823}	69.501	0.585839	newly	12.	3.54	{0., 6.}	79.	0.830631	Reff	2.91044	1.97435	{1.21382, 2.81653}	32.3969	ar	9371.	6046.06	{6., 9199.}	35.5219
40	R0	{2.40923, 15.1963, 0.1}	{2.26972, 10.233, 0.57}	{{1.47488, 4.06661, 0.}, {2.73634, 13.3345, 2.}}	{14.7959, 34.5023, 590.}	inf	149.	132.42	{52.2514, 193.299}	29.3133	0.168361	newly	86.	140.14	{92., 185.}	64.8837	0.205587	Reff	2.34731	2.2132	{1.45108, 2.65737}	14.4811	ar	8735.	8199.73	{5808., 9209.}	9.88655
41	R0	{2.63137, 15.238, 1.}	{2.11984, 13.4893, 1.22}	{{1.44485, 5.43578, 0.}, {2.64334, 19.255, 2.}}	{20.4419, 28.8044, 62.}	inf	88.	69.2784	{31.3145, 102.579}	31.8196	0.196367	newly	67.	52.96	{29., 81.}	39.3731	0.213645	Reff	2.59269	2.09156	{1.4328, 2.59967}	20.2495	ar	9150.	7805.8	{5390., 9091.}	14.8874
42	R0	{2.48769, 19.4593, 0.1}	{2.02498, 14.2262, 0.21}	{{1.78225, 11.7348, 0.}, {2.66225, 19.9254, 1.}}	{22.249, 28.1381, 270.}	inf	353.	207.056	{135.47, 363.618}	42.7757	0.274892	newly	145.	126.78	{91., 168.}	21.8069	0.105815	Reff	2.34714	1.93664	{1.71224, 2.51099}	21.1288	ar	8932.	7747.55	{7074., 9120.}	14.3384
43	R0	{2.61199, 10.214, 0.1}	{1.83756, 13.6345, 0.36}	{{1.1315, 6.2492, 0.}, {2.64721, 19.0665, 1.}}	{31.5627, 46.5403, 400.}	inf	1.	3.00967	{1.22765, 5.35747}	200.967	0.404276	newly	2.	3.19	{0., 6.}	122.5	0.456991	Reff	2.61147	1.83678	{1.13125, 2.64652}	31.5706	ar	9061.	5314.86	{3., 9149.}	41.9108
44	R0	{2.50994, 18.3382, 0.1}	{2.47377, 18.4459, 0.12}	{{1.78225, 11.7348, 0.}, {2.66225, 19.9254, 1.}}	{10.5435, 12.3954, 196.}	inf	194.	206.958	{109.973, 257.152}	23.6913	0.107007	newly	107.	102.07	{74., 123.}	13.5234	0.0633439	Reff	2.42761	2.38781	{1.73979, 2.56996}	10.0991	ar	8827.	8731.06	{7284., 9171.}	6.06661
45	R0	{2.44283, 16.8338, 0.1}	{2.33919, 12.6666, 0.83}	{{1.53501, 4.02511, 0.}, {2.98558, 18.1089, 2.}}	{17.3753, 30.1426, 800.}	inf	169.	142.751	{65.0323, 214.796}	29.272	0.188648	newly	105.	122.89	{74., 173.}	32.4286	0.124578	Reff	2.37394	2.27687	{1.50029, 2.88562}	17.0388	ar	8878.	8312.36	{6074., 9378.}	10.039
46	R0	{2.51815, 18.1462, 5.}	{2.34583, 15.1856, 0.32}	{{2.11132, 12.644, 0.}, {2.49092, 18.2812, 1.}}	{9.23199, 16.562, 93.6}	inf	163.	161.385	{110.68, 212.02}	20.7507	0.101222	newly	80.	101.81	{80., 128.}	30.6625	0.112498	Reff	2.44689	2.27877	{2.05644, 2.42607}	9.12876	ar	8978.	8574.3	{8136., 9040.}	5.1096
47	R0	{2.88885, 16.8987, 0.1}	{2.29949, 14.6596, 0.16}	{{1.81647, 10.5184, 0.}, {2.81891, 18.6082, 1.}}	{20.6738, 18.9099, 228.}	inf	49.	45.6239	{21.127, 70.4523}	29.7978	0.163662	newly	30.	29.86	{17., 43.}	30.4667	0.146521	Reff	2.86458	2.28038	{1.80244, 2.79872}	20.6444	ar	9337.	8341.84	{7361., 9308.}	10.706
48	R0	{2.58709, 13.8786, 0.1}	{2.33172, 10.7529, 0.96}	{{1.24751, 3.07157, 0.}, {2.98125, 17.1847, 2.}}	{18.6859, 33.1734, 940.}	inf	46.	31.3999	{7.89832, 55.9291}	42.05	0.308083	newly	26.	34.43	{11., 65.}	65.0385	0.240056	Reff	2.57002	2.31897	{1.24482, 2.96321}	18.6165	ar	9146.	7615.3	{2597., 9384.}	18.101
49	R0	{2.42959, 10.7132, 0.1}	{1.84962, 12.0503, 0.67}	{{1.14344, 6.00596, 0.}, {2.64334, 17.7287, 2.}}	{27.8078, 32.5281, 668.}	inf	18.	5.72092	{1.63096, 13.5985}	68.3707	0.61476	newly	13.	5.86	{1., 12.}	73.2308	0.684358	Reff	2.42424	1.84768	{1.14256, 2.63867}	27.7541	ar	8863.	5812.41	{10., 9076.}	35.5362
50	R0	{2.60513, 13.2796, 0.1}	{2.04633, 13.1913, 1.26}	{{1.28596, 4.25564, 0.}, {2.7499, 19.0355, 2.}}	{24.0622, 32.2966, 1206.}	inf	24.	21.0028	{7.98527, 37.8297}	43.04	0.23673	newly	29.	16.47	{4., 31.}	49.5517	0.369769	Reff	2.59445	2.03809	{1.28293, 2.73793}	24.0125	ar	9095.	7225.71	{3588., 9152.}	21.139
